# Photocatalytic Activity of Magnetic Nano-β-FeOOH/Fe_3_O_4_/Biochar Composites for the Enhanced Degradation of Methyl Orange Under Visible Light

**DOI:** 10.3390/nano11020526

**Published:** 2021-02-18

**Authors:** Zheng Zhang, Guanghua Wang, Wenbing Li, Lidong Zhang, Benwei Guo, Ling Ding, Xiangcheng Li

**Affiliations:** 1 School of Chemistry and Chemical Engineering, Wuhan University of Science and Technology, Wuhan 430081, China; zhangzheng@wust.edu.cn (Z.Z.); wangguanghua@wust.edu.cn (G.W.); zld950919@wust.edu.cn (L.Z.); guobenwei@wust.edu.cn (B.G.); dingling@wust.edu.cn (L.D.); 2 Research Center for Green and Intelligent Coal Chemical Engineering, Wuhan University of Science and Technology, Wuhan 430081, China; 3 The State Key Laboratory of Refractories and Metallurgy, Wuhan University of Science and Technology, Wuhan 430081, China

**Keywords:** graphene-like structure, β-FeOOH, photocatalysis, superparamagnetism

## Abstract

A novel nano-β-FeOOH/Fe_3_O_4_/biochar composite with enhanced photocatalytic performance and superparamagnetism was successfully fabricated via an environmentally friendly one-step method. The structural properties of the prepared composite were characterized by scanning electron microscopy, transmission electron microscopy, energy-dispersive spectroscopy, X-ray photoelectron spectroscopy, and a vibrating sample magnetometer. The XPS spectrum of the as-prepared composites confirmed the presence of Fe-O-C bonds between β-FeOOH and biochar, which could be conducive to transfer photo-generated electrons. UV-vis spectroscopy confirmed the existence of an electron–hole connection between β-FeOOH and biochar, which promoted the rapid interface transfer of photogenerated electrons from β-FeOOH to biochar. These novel structures could enhance the response of biochar to accelerate the photoelectrons under visible light for more free radicals. Electron spin resonance analysis and free radical quenching experiments showed that •OH was the primary active species in the photodegradation process of methyl orange by nano-β-FeOOH/Fe_3_O_4_/biochar. In the synergistic photocatalytic system, β-FeOOH/Fe_3_O_4_/biochar exhibited excellent catalytic activity for the degradation of azo dye (methyl orange), which is 2.03 times higher than that of the original biochar, while the surface area decreased from 1424.82 to 790.66 m^2^·g^−1^. Furthermore, β-FeOOH/Fe_3_O_4_/biochar maintained a stable structure and at least 98% catalytic activity after reuse, and it was easy to separate due to its superparamagnetism. This work highlights the enhanced photocatalytic performance of β-FeOOH/Fe_3_O_4_/biochar material, which can be used in azo dye wastewater treatment.

## 1. Introduction

Water pollution has further aggravated the contradiction of water shortage, among which azo dye wastewater is one of the primary harmful industrial wastewaters [1]. Methyl orange with azo bonds is classified as a difficult-to-handle organic dye because of its relatively high biological toxicity and difficult decomposition [2,3]. In view of the water pollution caused by azo dyes, there is an urgent need to develop effective treatment technologies. The traditional technologies used in azo dye wastewater treatment include adsorption [4,5], membrane filtration [6,7], biological methods [8], and advanced oxidation methods [9,10]. At present, the promising technology of wastewater treatment has focused on the characteristics of materials used in water treatment with the high removal performance, available feedstock, low cost, and easy operation for both adsorption and advanced oxidation methods [11,12]. The adsorption removal of pollutants in wastewater is simple, low-cost, high-speed, and high-efficiency, but the pollutants still cannot be eradicated [13,14]. The advanced oxidation process through free radicals generated by adding strong oxidants can degrade organic pollutants indiscriminately. However, these methods can lead to the secondary pollution problem for strong oxidants and adsorbents [15]. The photocatalytic method in the oxidation method can be green to degrade organic pollutants without adding an oxidant through the superoxide, and hydroxyl radicals generated by photosensitization nonselectively destroy the structure of the most refractory organic pollutants in wastewater [16,17]. 

The photocatalytic process does not need to add any oxidants except for the addition of photocatalysts and will not cause secondary pollution to the environment [18]. However, photocatalysts are still dominated by high-cost graphene materials, precious metals [19], and metal semiconductors [20]. Thus, there is an urgent need to develop an eco-friendly photocatalyst for potential application in the remediation of wastewater. Biochar has the characteristics of structures such as photocatalysts with graphene-like structures and carbon quantum dots that can be proved to stimulate the oxygen in water to generate hydroxyl radicals under visible light to degrade pollutants in our previous studies. Although the graphite-like structure on the biochar can promote the rapid transfer of photogenerated electrons [21], there is a synergy on the electron holes in the large π bond structure used as electron acceptors and donors to promote transfer carriers [22]. The enhancement of photocatalytic efficiency through the increase in the number of electron transfer channels should mainly accelerate the number of electrons transferred, which can be the reason for the better photocatalytic effect [23]. In order to improve the photocatalytic performance of biochar for more photogenerated electrons, nano-metal photocatalytic materials are introduced into the biochar to obtain the performance of a comprehensive response range and strong photocatalytic ability [24]. β-FeOOH loaded on biochar as a very common material due to its high oxidation activity, excellent qualitative properties, and low cost has electron holes that can obtain a strong visible light response in the process of the photodegradation of organic pollutants [25]. However, the synergistic mechanism of the enhancement of photocatalytic efficiency by the biochar with the structure of the graphene-like layer and carbon quantum dots is still unclear. 

Magnetic separation technology has been considered an effective method to separate solid-phase nanomaterials from heterogeneous systems [26]. Nano-iron oxide particles are superparamagnetic and can effectively separate them, especially because the crystallographic defect on the surface of Fe_3_O_4_ can promote the decomposition of hydrogen peroxide in the photocatalytic process to improve the photocatalytic process’ ability [27]. In addition, with its rich pore structure and abundant surface functional groups, biochar can be used as an excellent carrier to prevent the agglomeration effect of iron oxides [28]. 

In this study, a simple one-pot method was adopted to prepare a novel β-FeOOH/Fe_3_O_4_/biochar composite photocatalyst based on the photogenerated electrons effect by the organic combination among corn cob biochar, β-FeOOH, and Fe_3_O_4_. Water and oxygen are excited under visible light to produce green oxidants such as hydrogen peroxide to obtain photodegradation and magnetic separation. The novel photocatalysts were characterized by SEM, TEM, FTIR, XRD, XPS, Raman spectroscopy, UV-vis, EPR, and a vibrating sample magnetometer (VSM). The photocatalytic activity of magnetic nano-β-FeOOH/Fe_3_O_4_/biochar composites was investigated through the photodegradation of methyl orange. The synergistic photocatalysis of β-FeOOH/Fe_3_O_4_/biochar was also investigated, including the carbon quantum dots on the biochar, structure of β-FeOOH, photocatalytic performance under visible light, and the magnetic performance, which can enhance the photocatalytic efficiency by inhibiting the quenching of hydroxyl radicals. The nano-β-FeOOH/Fe_3_O_4_/biochar composites have potential prospects for application in the photodegradation of azo dye wastewater treatment.

## 2. Materials and Methods

### 2.1. Chemicals

The corn cob was collected from rural areas in Shandong, China. Ferrous chloride (FeCl_2_) and ferric chloride (FeCl_3_), sodium hydroxide (NaOH), ethanol, sodium dodecyl benzene sulfonate (SDBS), methyl orange (MO), and tert-butanol (TBA) were all supplied by China Pharmaceutical Reagent Co., Ltd. (Tianjin, China). All chemical reagents were of analytical grade.

### 2.2. Preparation of Catalysts

#### 2.2.1. Preparation of Biochar

The corn cob was crushed and ground, and then the corn cob powder was obtained by using a 100-mesh sieve. The biomass powder was then put into the corundum ark, heated to 600 °C at a temperature rising rate of 10 °C/min in a nitrogen atmosphere tube furnace, and then kept for two hours to cool to room temperature to obtain biochar powder.

#### 2.2.2. Preparation of β-FeOOH/Fe_3_O_4_/Biochar

β-FeOOH/Fe_3_O_4_ was synthesized by the one-step method. In short, 1 g of biochar was dispersed in 100 mL of deionized water, and then SDBS (1 g), FeCl_3_ (1.8 g), and FeCl_2_ (0.35 g) were dissolved in 50 mL of deionized water, and then mixed. After that, it was sonicated for 5 min, and then stirring was continued at 80 °C for 1 h gently. Then, 10 mL of aqueous ammonia solution was dropped into the mixture to stabilize the pH at 9. After aging the product for 2 h, it was allowed to cool naturally. Finally, the powder was collected by magnetic separation, repeatedly washed with deionized water and absolute ethanol, and dried overnight under vacuum at 50 °C to obtain 1.5 g of nano-β-FeOOH/Fe_3_O_4_/biochar composites. The mass ratio of biochar to iron oxide was 2:1, and the mass ratio of β-FeOOH to Fe_3_O_4_ in iron oxide was also 2:1.

### 2.3. Characterization of Catalysts

The structure of biochar and catalyst was tested and analyzed by an X-ray diffractometer (XRD, D8 Advance, Bruker, Karlsruhe, Germany), Cu target, Kα (λ = 0.154056 nm, U = 40 kV, I = 50 mA). Secondary scanning electron microscopy (SEM, Nava 400, FEI, Waltham, MA, USA) and transmission electron microscopy (TEM, JEM-100CXII, JEOL, Tokyo, Japan) were used to characterize the morphology of the catalyst, and the energy analyzer (EDX Nava 400, FEI, Waltham, MA, USA) was used for elemental analysis. Further, the N_2_ adsorption and desorption experiments were used to show the pores of the catalyst and structure, and calculate its specific surface area. The oxidation state of the elements in the biochar and catalyst was tested and identified by an X-ray photoelectron spectrometer (XPS, Multilab 2000, VG, Waltham, MA, USA). Fourier-transform infrared spectroscopy (FTIR) of the sample was carried out by a Nicolet 6700 Fourier Transform Infrared Spectrometer (Thermo Fisher, Waltham, MA, USA). The hysteresis loop of the composite catalyst was measured with a JDAW-2000D vibrating sample magnetometer (VSM, Yingpu Corp., Shanghai, China).

### 2.4. Degradation of MO

In the photocatalytic degradation experiment, 0.1 g of the catalyst was utterly dispersed in 100 mL of 100 mg/L MO solution, and the mixture was magnetically stirred in a dark environment at 25 °C to have it shaded and adsorbing for 4 h. The pH value of the reaction was 5.6, which is the initial value of the methyl orange solution. After that, the 350 W xenon lamp with a 420 nm cut-off filter mimicked visible light for degradation experiments. In a certain interval, a pipette was used to remove a sample of the solution and immediately magnetically separate the supernatant from obtaining the supernatant, and the MO concentration was measured with a spectrophotometer. The concentration of methyl orange was detected by a spectrophotometer at a wavelength of 463 nm. In the control experiment, only one variable was changed at a time. In the free radical detection experiment, tert-butanol was added to the solution. For repeated experiments of photocatalytic degradation, the spent catalyst was collected by magnetic separation after the degradation experiment and washed with deionized water and ethanol three times before the next test run. After that, the catalyst was dispersed in a system with the same parameters. All experiments were repeated three times, the average value was taken, and an error bar analysis chart was made.

## 3. Results and Discussion

### 3.1. Structural Analysis of Biochar and Composite Catalyst

**Figure 1:**
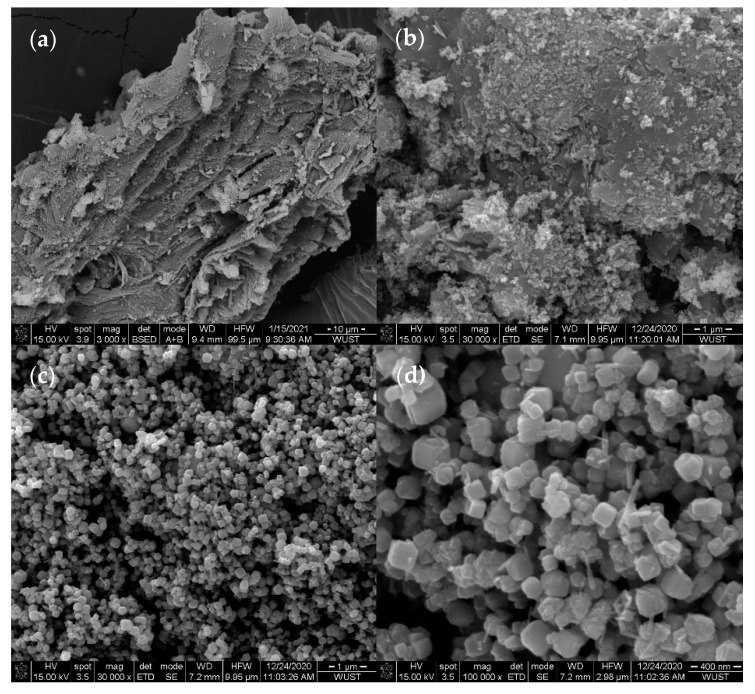
SEM images of biochar and β-FeOOH/Fe_3_O_4_/biochar: (**a**,**b**) Biochar. (**c**,**d**) β-FeOOH/Fe_3_O_4_/biochar.

It can be seen from the SEM images that the corn cob biochar in Figure 1a,b shows that the corn cob biochar has a unique pore structure and contains a graphene-like layer structure, which can provide active sites for the loading of β-FeOOH and Fe_3_O_4_. It can be seen from Figure 1c,d that the catalyst is attached to the surface of the biochar in the form of nanoparticles and nanorods, and the loading of the catalyst does not change the morphology of the biochar. Among them, Fe_3_O_4_ is loaded on biochar with nanoparticle sizes of 15–30 nm, and β-FeOOH is loaded on biochar in a rod shape [27].

**Figure 2:**
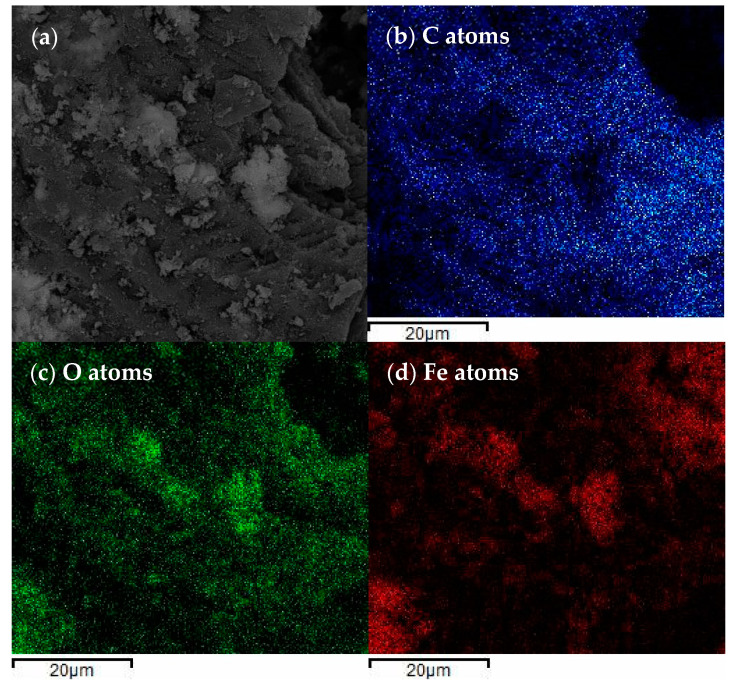
SEM and EDX images of β-FeOOH/Fe_3_O_4_/biochar: (**a**) SEM image of β-FeOOH/Fe_3_O_4_/biochar. (**b**) C atoms distribution. (**c**) O atoms distribution. (**d**) Fe atoms distribution.

The spatial distribution of elements, dispersive energy X-ray (EDX) spectrum, and element mapping is shown in Figure 2, where Figure 2b shows the distribution of C atoms, Figure 2c shows the distribution of O atoms, and Figure 2d represents the distribution of Fe atoms. It can be seen from the figure that the C, O, and Fe atoms are uniformly distributed, indicating that the catalyst is evenly combined with biochar, which also corresponds to the SEM results [29].

**Figure 3:**
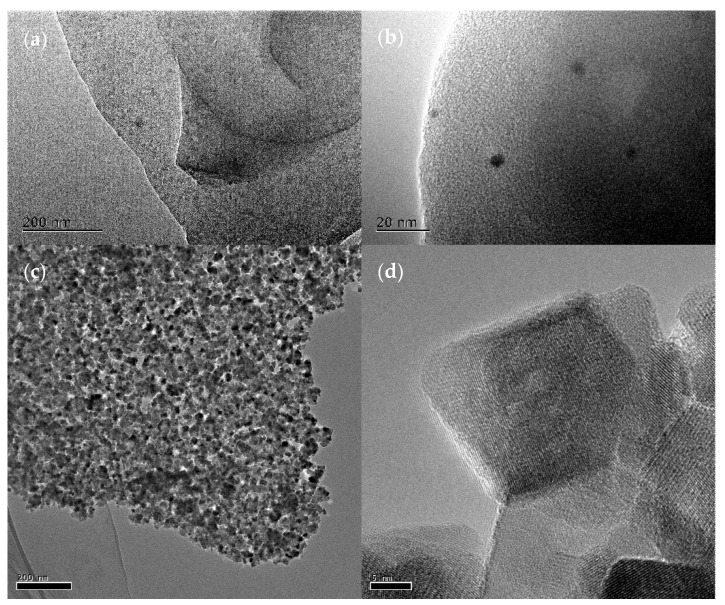
TEM images of biochar and β-FeOOH/Fe_3_O_4_/biochar: (**a**,**b**) TEM images of biochar. (**c**,**d**) TEM images of β-FeOOH/Fe_3_O_4_.

It can be seen from Figure 3a that the biochar is gradually graphitized, and the graphene-like layered structure can be seen under TEM at the edges. Moreover, in Figure 3b, it can be seen that biochar has carbon quantum dots, which provides a guarantee for the photocatalytic performance of biochar and the hybridization of β-FeOOH quantum dots. It can be seen from Figure 3c,d that β-FeOOH/Fe_3_O_4_ shows the prepared TEM image, which indicates that biochar has successfully combined with β-FeOOH/Fe_3_O_4_.

**Figure 4:**
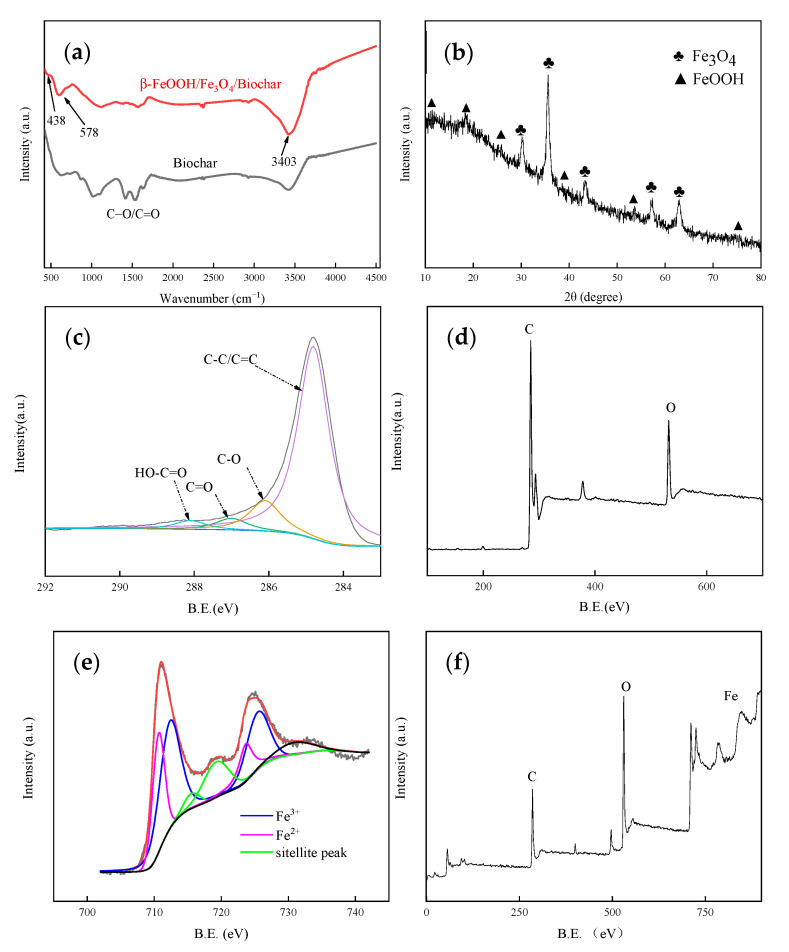
(**a**) FTIR spectra analysis image. (**b**) XRD spectra analysis image. (**c**,**d**) XPS spectral analysis image of biochar. (**e**,**f**) XPS spectra analysis image of β-FeOOH/Fe_3_O_4_/biochar.

The FTIR spectra of Biochar and β-FeOOH/Fe_3_O_4_/biochar are shown in Figure 4a. Near 3403 cm^−1^, both biochar and composite materials have firm peaks [30]. Generally, the absorption band near 3410 cm^−1^ is attributed to the vibration of OH in β-FeOOH. The peak in biochar indicates that it contains phenolic hydroxyl groups. The reason for the decrease in the composite material peaks is that β-FeOOH and Fe_3_O_4_ combine with part of the phenolic hydroxyl groups during the loading process, and the peak of biochar decreases at 1250~1750 cm^−1^. This proves that β-FeOOH and Fe_3_O_4_ are successfully combined with oxygen-containing functional groups on biochar. The typical low-frequency band of β-FeOOH and Fe_3_O_4_ samples near 578 cm^−1^ involves Fe^III^-O vibrations at the parts, and the 429 cm^−1^ in the Fe_3_O_4_ sample is attributed to the Fe^II^-O vibrations at the octahedral position. The band of β-FeOOH in the composite material at 578 cm^−1^ confirms the connection of β-FeOOH/Fe_3_O_4_ and biochar. As shown in Figure 4b, the peak position of β-FeOOH/Fe_3_O_4_ is shown in the figure. The peak intensity of β-FeOOH is weaker than that of Fe_3_O_4_, which is caused by the insignificant vibration of Fe III-O in β-FeOOH. All diffraction peaks of the synthesized solid conform to the standard data of the Fe_3_O_4_ face-centered-cubic crystal and tetragonal β-FeOOH, which correspond to the results of SEM and TEM.

XPS spectroscopy is used to detect the oxidation state of various elements in the sample and further analyze the composition and valence of the sample surface elements. Scanning XPS spectra of biochar and β-FeOOH/Fe_3_O_4_/biochar composites shown in Figure 4c,e show that C and O elements are present both samples, but only the β-FeOOH/Fe_3_O_4_/Fe element can be observed in biochar. The corresponding high-resolution XPS spectra of biochar are shown in Figure 4c,d. The two characteristic binding energies of C-O and C=O from biochar appear at 159.16 and 164.47 eV, respectively, and the spin–orbit splitting is 5.31 eV. As shown in Figure 4e,f, the characteristic peaks of Fe^2+^ and Fe^3+^ are at 711.60 and 725.50 eV, and 718.83 eV, respectively. Compared with the average amounts of Fe_3_O_4_ and β-FeOOH, the Fe^2+^ in the composite ratio is higher than β-FeOOH and lower than Fe_3_O_4_. After β-FeOOH/Fe_3_O_4_ is deposited, the C-O binding energy of the sample β-FeOOH/Fe_3_O_4_/biochar remains unchanged, which indicates that β-FeOOH/Fe_3_O_4_ is located on the surface of the biochar and is not integrated into the crystal lattice through the formation of chemical bonds [31].

**Figure 5:**
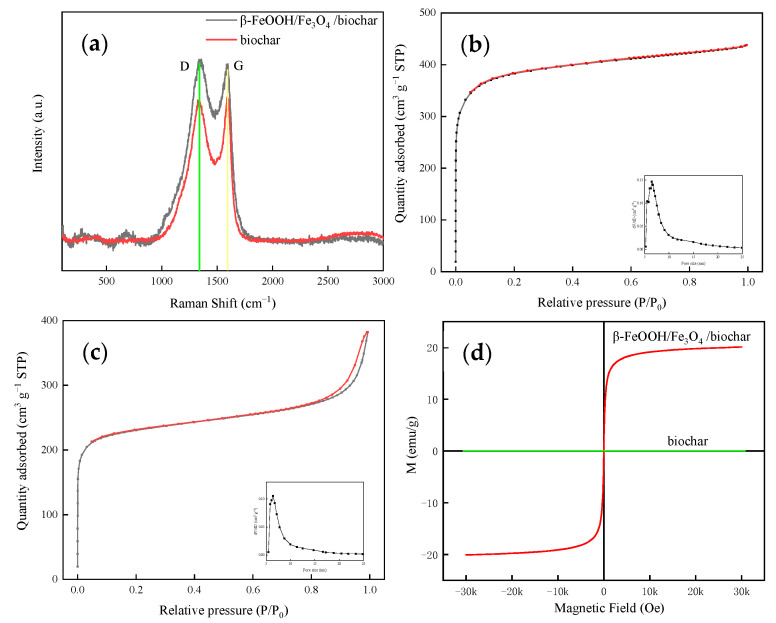
(**a**) Raman spectral analysis image. (**b**) Nitrogen adsorption–desorption isotherms of biochar; the inset is the pore size distribution. (**c**) Nitrogen adsorption–desorption isotherms of β-FeOOH/Fe_3_O_4_/biochar; the inset is the pore size distribution. (**d**) Vibrating sample magnetometer (VSM) pattern of β-FeOOH/Fe_3_O_4_/biochar.

The Raman spectra confirmed structural changes in biochar with respect to the composite. As shown in Figure 5a, biochar shows two peaks at 1339 and 1587 cm^−1^, which are the D band and the G band. The Raman peaks of the composite are widened and the baseline has been raised, comparing with the Raman spectrum of pristine biochar. Compared with biochar, the increase in the intensity ratio (ID/IG) of the D band and the G band confirms the reduction in the carbon sp^2^ domain. Clear G and D bands are observed in the Raman spectrum, which confirms the formation of β-FeOOH/Fe_3_O_4_ on the graphene-like structure. It is obvious that the β-FeOOH/Fe_3_O_4_ nanoparticles inlay in the graphene-like structure and the grain size of Fe nanoparticles ranges from 10 to 50 nm.

The tissue structure of biochar and the connection structure of β-FeOOH/Fe_3_O_4_/biochar can be determined by the nitrogen adsorption–desorption isotherms shown in Figure 5b,c, respectively. As shown in the figure, this means that the aggregated particles form an irregular mesoporous structure. The specific surface area of biochar is 1424.82 m^2^·g^−1^, and the specific surface area of β-FeOOH/Fe_3_O_4_/biochar is 790.66 m^2^·g^−1^, which also indicates that the combination of β-FeOOH/Fe_3_O_4_/biochar will occupy part of the active surface sites, resulting in a decrease in the specific surface area. The existence of a high surface area and the high proportion of mesopores can provide abundant active sites, which can shorten the transfer path of photogenerated electrons, thereby improving the photocatalytic performance of β-FeOOH/Fe_3_O_4_/biochar composites.

As shown in Figure 5d, the VSM hysteresis loop’s detection line shows that the prepared composite catalyst’s detection line passes through the origin, indicating that it has superparamagnetism. However, biochar has almost no magnetism, which indicates that the source of magnetism in the β-FeOOH/Fe_3_O_4_/biochar composite is Fe_3_O_4_. Compared with ordinary filtration and separation, superparamagnetism makes the catalyst easier to separate and more efficient [32,33].

### 3.2. Adsorption Experiment

**Figure 6:**
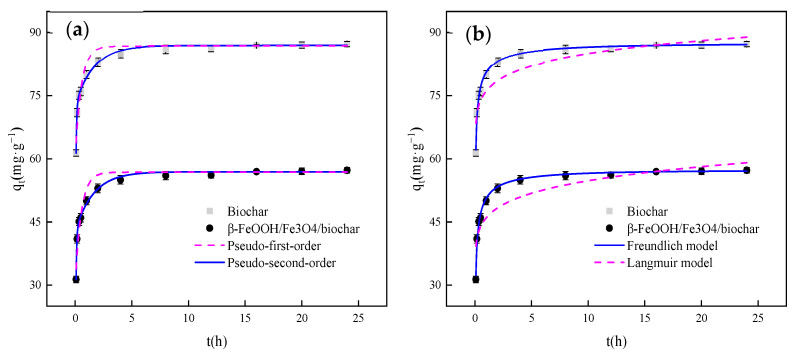
(**a**) Adsorption experiment and nonlinear fitting of dynamics, (**b**) adsorption experiment and nonlinear fitting of thermodynamics.

Figure 6a,b are the results of shading adsorption of biochar and β-FeOOH/Fe_3_O_4_/biochar composites. In the shading adsorption experiment, the adsorption capacity of biochar is better than that of the catalyst. First, in the same quality of β-FeOOH/Fe_3_O_4_, the mass of biochar only accounts for two-thirds of the total mass. Second, when the β-FeOOH/Fe_3_O_4_ is loaded on the biochar, the β-FeOOH/Fe_3_O_4_ will block the pores on the biochar or occupy the oxygen-containing functional groups on its surface. This can also be directly reflected in the results of the specific surface area.

#### 3.2.1. Kinetic Experiment

In the adsorption kinetic experiment, 100 mL of methyl orange solution is taken with an initial concentration of 100 mg/L, 0.1 g of adsorbent is added, a series of reaction time gradients are set, and the concentration of methyl orange is measured after adsorption from 0 to 24 h. The pseudo first-order adsorption kinetic model and pseudo-secondary adsorption kinetic model are commonly used to analyze the adsorption process and calculate the adsorption rate [34].

The pseudo-first-order kinetic equation is expressed in Equation (1):

(1) $$ln(Q_{e}-Q_{t})=lnQ_{e}-k_{1}t$$

where *Q*_e_ is the amount of adsorption at adsorption equilibrium, mg/g; *Q*_t_ is the amount of adsorption at time *t*, mg·g^−1^; *k*_1_ is the rate constant of the model equation, min^−1^. 

The pseudo-second-order kinetic equation is expressed in Equation (2):

(2) $$\frac{t}{Q_{t}}=\frac{1}{k_{2}Q_{e}^{2}}+\frac{1}{Q_{e}}t$$

where *k*_2_ is the rate constant of the model equation, g·(mg·min)^−1^. 

According to Figure 6a and Table 1, the adsorption of MO by biochar and β-FeOOH/Fe_3_O_4_/biochar is fitted with a pseudo-second-order kinetic model. The linear correlation coefficient is 0.99, which is similar to the pseudo-first-order kinetic model. Compared with the correlation coefficient obtained by fitting 0.87, the fitting effect is better. The pseudo-second-order kinetic equation also shows that the sharing or exchange of free electrons occurs during the adsorption process, and chemical adsorption involving covalent forces occurs. In β-FeOOH/Fe_3_O_4_/biochar, the combination of β-FeOOH and carbon quantum dots will reduce the adsorption performance, but can enhance the ability of electron sharing.

#### 3.2.2. Thermodynamic Experiment 

In the adsorption isotherm experiment, 20 mL of a series of the concentration gradients (50–300 mg/L) of methyl orange solution was taken, 20 mg of adsorbent was added, and the adsorption time was set to 480 min. The commonly used adsorption isotherm models are the Langmuir and Freundlich models. The Langmuir model is based on the adsorption of adsorbates on the surface of the adsorbent in a single molecular layer, and Freundlich is based on the adsorption on heterogeneous surfaces. Langmuir and Freundlich isotherm model fitting analysis was performed on the data obtained from the adsorption experiment [35].

The Langmuir isothermal equation is expressed in Equation (3):

(3) $$\frac{C_{e}}{Q_{e}}=\frac{C_{e}}{Q_{m}}+\frac{1}{K_{L}Q_{m}}$$

where *C*_e_ is the concentration when the adsorption reaches equilibrium, mg·L^−1^; *Q*_e_ is the amount of adsorption when the adsorption reaches equilibrium, mg·g^−1^; *Q*_m_ is the maximum adsorption, mg/g; *K*_L_ is the adsorption equilibrium constant, L·g^−1^;

The Freundlich isotherm equation is expressed as Equation (4):

(4) $$lnq_{e}=lnK_{F}+\frac{1}{n}lnC_{e}$$

where *K*_F_ [(mg·g^−1^) (L·mg^−1^)^1/n^] is the Freundlich capacity constant, and *n* is the Freundlich intensity constant. 

The linear and nonlinear fitting results of Langmuir and Freundlich adsorption isotherm equations can be obtained from Figure 6b and Table 2. The 1/n value of the adsorption constant according to the Freundlich model can be used to determine the difficulty of adsorption. If l/n > 2, the adsorption is not easy. If 1/n is between 0.1 and 0.5, the adsorption reaction is easier to proceed. From the analysis results in the table, the adsorption reaction is easy if 1/n is between 0.2 and 0.3.

### 3.3. Photocatalytic Degradation Performance 

#### 3.3.1. Adsorption Performance

**Figure 7:**
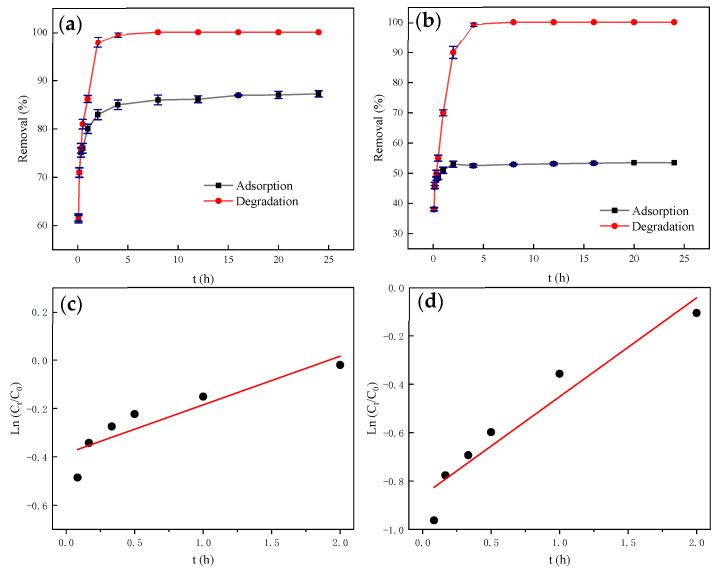
(**a**) Experiment of shading adsorption and photocatalytic degradation of biochar. (**b**) Experiment of shading adsorption and photocatalytic degradation of β-FeOOH/Fe_3_O_4_/biochar. (**c**) Kinetic analysis of photocatalytic degradation experiment of biochar. (**d**) Kinetic analysis of photocatalytic degradation experiment of β-FeOOH/Fe_3_O_4_/biochar.

Figure 7a,b show the shading adsorption results and visible light photocatalytic degradation of biochar and β-FeOOH/Fe_3_O_4_/biochar composites, respectively. Under light-shielding adsorption conditions, biochar and β-FeOOH/Fe_3_O_4_/biochar composites’ adsorption capacity when adsorbing the same concentration of methyl orange solution is significantly different. The reason is that when the catalyst is loaded, some active sites on the biochar are occupied, which can be obtained from the FTIR diagram. Furthermore, in the BET results, it can be seen that as the catalyst is loaded, the catalyst particles may block some delicate pore structures, resulting in a significant decrease in adsorption capacity.

#### 3.3.2. Performance of Photocatalytic

Under the same conditions, due to the excellent adsorption effect of pure biochar, the biochar degradation system has a good treatment effect within one hour in the beginning. Then, over time, the catalyst has a good effect on MO due to its excellent photocatalytic properties. The processing capacity gradually increases, and the final degradation time is faster than that of biochar [36].

The degradation kinetics is used to indicate the degradation rate constant of methyl orange in the photocatalysis experiment, and the formula is as follows:

(5) $$\ln\left(C_{t}/C_{0}\right)=kt$$

where *k* is the rate constant of photocatalytic degradation; *t* is the moment of photocatalytic reaction, min^−1^; *C*_t_ is the MO equilibrium content at time t, mg·L^−1^; *C*_0_ is the MO content before the reaction, mg·L^−1^. 

Figure 7c,d show the linear fitting graphs of ln(*C*_t_/*C*_0_) of biochar and β-FeOOH/Fe_3_O_4_/biochar versus *t*, where the slope of the fitting line is the degradation rate constant *k*. As shown in Table 3, the photodegradation *k* of β-FeOOH/Fe_3_O_4_/biochar is 2.03 times that of biochar photodegradation. From the perspective of degradation rate, β-FeOOH/Fe_3_O_4_/biochar can generate more hydroxyl radicals faster, which indicates that hydroxyl radicals play an essential role in the degradation of methyl orange.

When β-FeOOH/Fe_3_O_4_ is loaded on biochar, it can improve the photocatalytic performance of biochar. The reasons for this situation may be the following: First, the graphene-like structure on biochar has a π–π conjugated structure, which is rich in electrons and holes. When combined with β-FeOOH/Fe_3_O_4_, they will form a Fe-O-C structure. This structure quickly flows photogenerated electrons from β-FeOOH/Fe_3_O_4_ to the graphene-like structure. Therefore, the separation efficiency and photocatalytic activity of electron–hole pairs are improved. Secondly, some photogenerated electrons also directly react with oxygen and H_2_O_2_ adsorbed on β-FeOOH and Fe_3_O_4_ to form hydroxyl radicals, thereby further improving the ability of biochar to absorb and convert visible light [37].

### 3.4. Research on Photocatalytic Mechanism of Catalyst

**Figure 8:**
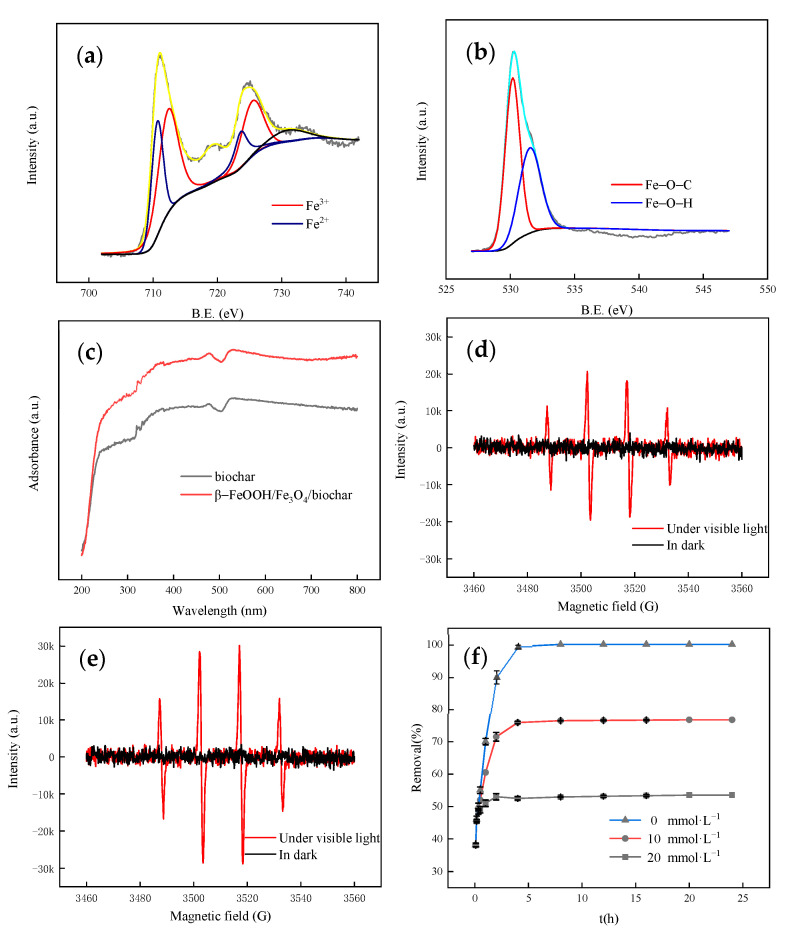
(**a**,**b**) XPS spectra analysis image of β-FeOOH/Fe_3_O_4_/biochar. (**c**) UV-vis diagrams. (**d**) EPR diagrams of biochar. (**e**) EPR diagrams of β-FeOOH/Fe_3_O_4_/biochar. (**f**) Free radical quenching experiment.

To determine why the combined action of β-FeOOH/Fe_3_O_4_/biochar can significantly improve the degradation of methyl orange, XPS was used to analyze the connection state between the C, O, and Fe of biochar and β-FeOOH/Fe_3_O_4_/biochar. The results were obtained and are given in Figure 8a,b. According to the XPS spectrum of 2p, the peaks appearing at 711.6 and 725.2 eV correspond to the 2p 3/2 and 2p 1/2 of Fe(III), and the peaks appearing at 710.3 and 723.7 eV corresponding to Fe(II) 2p 3/2 and 2p 1/2 indicate that β-FeOOH and Fe_3_O_4_ phases are formed in the composite. The binding energy position of β-FeOOH/Fe_3_O_4_/biochar is positively shifted by 0.2 eV. The two prominent peaks are located at 712 and 726 eV, indicating an electronic interaction between biochar and β-FeOOH/Fe_3_O_4_. The O1s spectrum of the composite material contains two characteristic peaks in Figure 4c. The peak at 531.2 eV indicates the C-O-iron bond, which implies that the biochar and FeOOH are connected by the C-O-Fe bond. XPS analysis and FT-IR analysis echo each other. It is confirmed that the existence of Fe-O-C bonds can inhibit the recombination of photo-generated electron–hole pairs on biochar, thereby improving light absorption and photocatalysis. 

UV-Vis diffuse spectroscopy (UV-Vis-DRS) was used to study the prepared samples’ light absorption characteristics. Figure 8c shows the UV-Vis-DRS of biochar and the β-FeOOH/Fe_3_O_4_/biochar composite. Note that compared with pure biochar, the edge of the absorption band of β-FeOOH/Fe_3_O_4_/biochar is shifted to low energy (redshift phenomenon), which indicates that the introduction of β-FeOOH/Fe_3_O_4_ can expand the visible light response range of biochar and increase the visible light utilization efficiency. The calculated band gap energies of biochar and β-FeOOH/Fe_3_O_4_/biochar are 2.66 and 2.36 eV, respectively, which indicates that the β-FeOOH/Fe_3_O_4_ deposited on the biochar carbon quantum dots helps to broaden the visible light response range of the catalyst and ultimately enhance photocatalytic activity. By confirming the visible light response intensity and photogenerated electron transfer in the studied materials, the materials’ photoelectric properties were evaluated. β-FeOOH as a photocatalytic sensitive material has a wide spectral absorption range, which can greatly improve biochar’s visible light absorption capacity. The light absorption characteristics of biochar and β-FeOOH/Fe_3_O_4_/biochar composites were compared. The enhanced light absorption capacity can better capture photoelectrons, thereby improving the conversion efficiency and transfer capacity of photogenerated electrons, thereby enhancing the photocatalytic degradation of methyl orange.

It can be seen from Figure 8d,e that both biochar and β-FeOOH/Fe_3_O_4_/biochar can produce a characteristic peak of 1:2:2:1 under visible light. This characteristic peak indicates that biochar and β-FeOOH/Fe_3_O_4_/biochar generated under visible light are hydroxyl radicals. Among them, β-FeOOH/Fe_3_O_4_/biochar is stronger than the characteristic peaks produced by biochar, which indicates that β-FeOOH/Fe_3_O_4_/biochar can transfer photo-generated electrons and generate free radicals faster under visible light, which improves the photocatalytic efficiency.

As a common hydroxyl radical scavenger, tert-butanol can qualitatively illustrate the existence of •OH. In the photocatalysis experiment, different concentrations of tert-butanol were added as a control experiment, as shown in Figure 8f, the experiment was carried out under visible light, and the other conditions were the same as the previous photocatalysis experiment conditions. The experimental results clearly show that the photocatalytic degradation reaction is inhibited after adding tert-butanol. When the concentration of tert-butanol continues to increase to 20 mg/L, there is no degradation reaction in the whole system. The possible reason is that the concentration of tert-butanol is so high that it completely quenches the hydroxyl radicals to inhibit the degradation reaction. It can be seen that the generation of hydroxyl radicals is the main degradation factor for the photo-induced degradation of methyl orange.

In the process of photocatalysis, photo-generated electrons e^–^ and photo-generated holes h^+^ will recombine to produce photons hv. It promotes photogenerated electrons’ transfer from the conduction band of β-FeOOH to the empty orbital of the graphene-like structure and carbon quantum dots through conjugated π-bond molecules [38]. It produces directional motion on the biochar to avoid simple recombination with photogenerated holes h^+^. The photo-generated holes [39] h^+^ have strong oxidizing properties and react with water molecules to generate •OH and H^+^. The photogenerated electron e can stimulate the graphene-like structure and the carbon quantum dots to react with oxygen in the water to make it negatively charged to produce •$O_{2}^{-}$ and CR^−^. The charged graphene-like structure can interact with oxygen molecules in the water to produce oxidizing H_2_O_2_. H_2_O_2_ can further interact with H^+^ and photo-generated electrons e^−^ or •$O_{2}^{-}$. Simultaneously, H_2_O_2_ can also be excited and converted into ·OH by absorbing the photon. •$O_{2}^{-}$ generated by the excitation of the photogenerated electron e can react with H^+^ to generate H_2_O_2_. So far, through the above reaction [40], strong oxidizing oxidants such as h+, •OH, and •$O_{2}^{-}$ can be produced, which oxidize the target degradation products in the solution to carbon dioxide and water. The reaction equation is shown in Equations (6)–(12):

(6) $$\mathrm{Fe}-O-C\;+\;hv\to e^{-}+\;h^{+}$$

(7) $$O_{2}+\;e^{-}\to \;•O_{2}^{-}$$

(8) $$h^{+}+\;H_{2}O\to H^{+}+•OH$$

(9) $$•O_{2}^{-}+\;H^{+}\to HO_{2}•$$

(10) $$e^{-}+CR\to CR•$$

(11) $$2CR•+O_{2}+2H^{+}\to 2CR+H_{2}O_{2}$$

(12) $$Fe_{3}O_{4}+H_{2}O_{2}+2e^{-}\to Fe_{3}O_{4}+•OH+OH^{-}$$

### 3.5. Reused Performance of β-FeOOH/Fe_3_O_4_/Biochar

**Figure 9:**
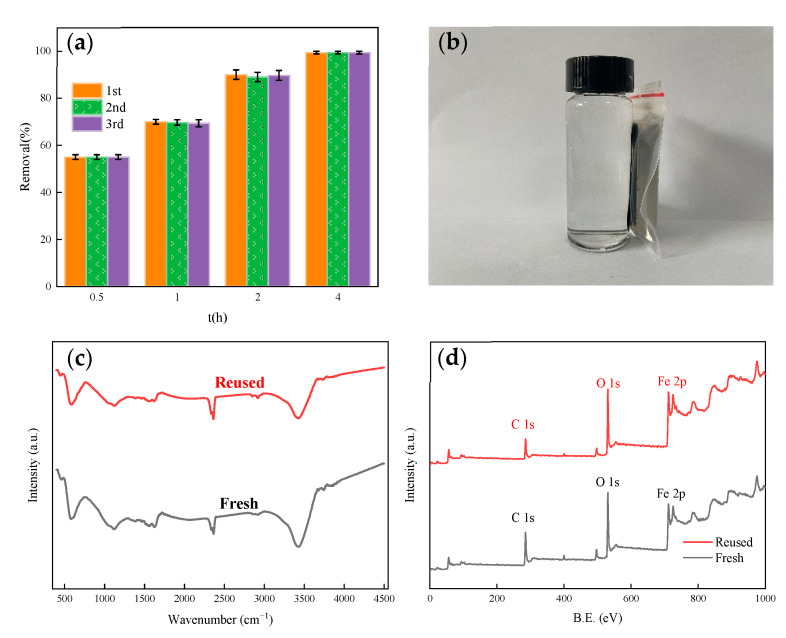
(**a**) Recycling experiment. (**b**) Magnetic separation image of β-FeOOH/Fe_3_O_4_/biochar. (**c**) FTIR diagram before and after use. (**d**) XPS diagram of before and after use.

It can be seen from Figure 9a that under the condition that other conditions are not changed, the photocatalytic effect of β-FeOOH/Fe_3_O_4_/biochar photocatalyst is still 98% of the first time after repeated use for three times. This shows that the β-FeOOH/Fe_3_O_4_/biochar catalyst has a good repeated-use effect and can still maintain high-efficiency photocatalytic performance after repeated use. It can be seen from Figure 9b that the magnet can separate β-FeOOH/Fe_3_O_4_/biochar in a very short time, which also shows that β-FeOOH/Fe_3_O_4_/biochar is very magnetic. It can also be seen from the previous VSM data that the composite is superparamagnetic [33,41].

It can be seen from Figure 9c,d in the FTIR and XPS diagrams of β-FeOOH/Fe_3_O_4_/biochar before use and after the third use that the structure of β-FeOOH/Fe_3_O_4_/biochar has no special effects, which also verifies that β-FeOOH/Fe_3_O_4_/biochar can maintain excellent photocatalytic activity after reuse [42].

## 4. Conclusions

A superparamagnetic nano-β-FeOOH/Fe_3_O_4_/biochar composite catalyst was prepared through a simple and feasible one-step method. XPS spectroscopy confirmed the formation of a stable Fe–O–C structure, and β-FeOOH and Fe_3_O_4_ could be fixed on biochar by the images of SEM and EDS. UV-vis spectroscopy confirmed the existence of an electron–hole connection between β-FeOOH and biochar, which promoted the rapid interface transfer of photogenerated electrons from β-FeOOH to biochar. These novel structures could enhance the response of biochar to accelerate the photoelectrons under visible light for more free radicals. In the synergistic photocatalytic system, β-FeOOH/Fe_3_O_4_/biochar exhibited excellent catalytic activity for the degradation of azo dye (methyl orange), which is 2.03 times higher than that of the original biochar while the surface area decreased from 1424.82 to 790.66 m^2^·g^−1^. Furthermore, β-FeOOH/Fe_3_O_4_/biochar maintained a stable structure and at least 98% catalytic activity after reuse and was easy to separate due to its superparamagnetism. Therefore, nano-β-FeOOH/Fe_3_O_4_/biochar, as an efficient and green heterogeneous photocatalyst, has greater potential application prospects in the photocatalytic treatment of organic wastewater.

## Figures and Tables

**Table 1:** Adsorption kinetic parameters.

Sample	Pseudo-Second-Order Kinetic Model			
	*Q_e_* (exp) (mg·g^−1^)	*k*_2_ (g·mg^−1^·min^−1^)	*Q_e_* (cal) (mg·g^−1^)	R^2^
Biochar	86.15	0.2316 ± 0.0421	88.28	0.99
β-FeOOH/Fe_3_O_4_/biochar	57.93	0.1645 ± 0.0352	58.11	0.99

**Table 2:** Adsorption isotherm parameters.

Sample	Freundlich Isotherm Model		
	*K_F_* (mg·g^−1^) (L·mg^−1^)1/n	*n*	R^2^
Biochar	9.9132 ± 0.0751	0.26	0.99
β-FeOOH/Fe_3_O_4_/biochar	6.4634 ± 0.0482	0.25	0.99

**Table 3:** Kinetic parameters of photocatalytic degradation.

Sample	*k*	R^2^
Biochar	0.2012 ± 0.0457	0.82
β-FeOOH/Fe_3_O_4_/biochar	0.4087 ± 0.0586	0.92

## Data Availability

The data presented in this study are available within this article. Further inquiries could be directed to the authors.
